# (1*RS*,4*RS*,5*RS*)-Methyl 2-(3,5-dinitro­benzo­yl)-2-oxa-3-aza­bicyclo­[3.3.0]oct-7-ene-4-carboxyl­ate

**DOI:** 10.1107/S160053680901246X

**Published:** 2009-04-08

**Authors:** Carlos A. D. Sousa, José E. Rodríguez-Borges, M. Luísa C. Vale, Xerardo Garcia-Mera

**Affiliations:** a Centro de Investigação em Química, Departamento de Química, Faculdade de Ciências, Universidade do Porto, Rua do Campo Alegre, 687, 4169-007 Porto, Portugal; b Departamento de Química Orgánica, Facultade de Farmacia, Universidade de Santiago de Compostela, E-15782 Santiago de Compostela, Spain

## Abstract

The title compound, C_15_H_13_N_3_O_8_, comprises two crystallographically independent mol­ecules in the asymmetric unit. In the crystal, intermolecular C—H⋯O hydrogen bonds link the molecules and short C=O⋯π contacts are seen.

## Related literature

For the preparation of the precursor of the title compound, see: Sousa *et al.* (2008 ▶). For examples of the use of the 3,5dinitro­benzoyl­ation technique for the assignment of structures by X-ray, see: Caamaño *et al.* (2000 ▶); Fernández *et al.* (2001 ▶). 
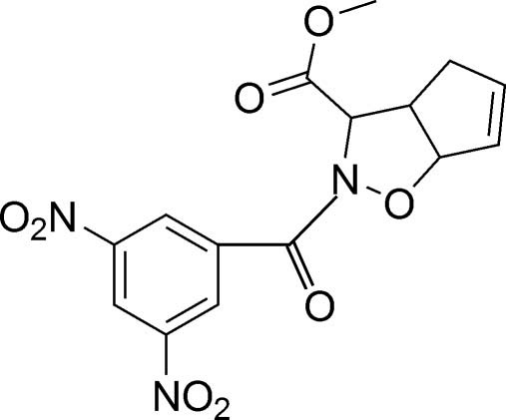


## Experimental

### 

#### Crystal data

C_15_H_13_N_3_O_8_
*M* *_r_* = 363.28
Triclinic, 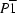

*a* = 8.7157 (3) Å
*b* = 10.8269 (3) Å
*c* = 17.0677 (5) Å
α = 79.881 (1)°
β = 77.773 (1)°
γ = 78.281 (1)°
*V* = 1526.35 (8) Å^3^
*Z* = 4
Mo *K*α radiation
μ = 0.13 mm^−1^
*T* = 100 K
0.26 × 0.23 × 0.1 mm

#### Data collection

Bruker ApexII CCD area-detector diffractometer
Absorption correction: multi-scan (*SADABS*; Bruker, 2007 ▶) *T*_min_ = 0.913, *T*_max_ = 0.99
28142 measured reflections
6012 independent reflections
4564 reflections with *I* > 2σ(*I*)
*R*_int_ = 0.039

#### Refinement

*R*[*F* ^2^ > 2σ(*F* ^2^)] = 0.047
*wR*(*F* ^2^) = 0.124
*S* = 1.05
6012 reflections
471 parameters
H-atom parameters constrained
Δρ_max_ = 0.27 e Å^−3^
Δρ_min_ = −0.24 e Å^−3^

### 

Data collection: *APEX2* (Bruker, 2007 ▶); cell refinement: *SAINT* (Bruker, 2007 ▶); data reduction: *SAINT*; program(s) used to solve structure: *SIR97* (Altomare *et al.*, 1997 ▶); program(s) used to refine structure: *SHELXL97* (Sheldrick, 2008 ▶); molecular graphics: *ORTEP-3 for Windows* (Farrugia, 1997 ▶); software used to prepare material for publication: *WinGX* publication routines (Farrugia, 1999 ▶).

## Supplementary Material

Crystal structure: contains datablocks I, global. DOI: 10.1107/S160053680901246X/kp2206sup1.cif

Structure factors: contains datablocks I. DOI: 10.1107/S160053680901246X/kp2206Isup2.hkl

Additional supplementary materials:  crystallographic information; 3D view; checkCIF report

## Figures and Tables

**Table 1:** Hydrogen-bond geometry (Å, °)

*D*—H⋯*A*	*D*—H	H⋯*A*	*D*⋯*A*	*D*—H⋯*A*
C17—H17⋯O11^i^	0.98	2.40	3.064 (3)	124
C47—H47⋯O41^ii^	0.98	2.45	3.083 (3)	122

Symmetry codes: (i) 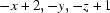; (ii) 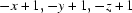.

**Table 2:** Geometric parameters of *Y*—*X*⋯*Cg* contacts (Å, °)

*Y*—*X*⋯*Cg*	*X*⋯*Cg*	*Y*—*X*⋯*Cg*	*Y*⋯*Cg*
C13—O14⋯*Cg*2	3.2009 (18)	106.47 (13)	3.736 (3)
C43—O44⋯*Cg*1	3.1434 (18)	104.52 (13)	3.649 (3)

*Cg*1 and *Cg*2 are the centroids of the rings defined by C1, C2, C3, C7, C8, C12 and C31, C32, C33, C37, C38, C42, respectively.

**Fig. 1.**
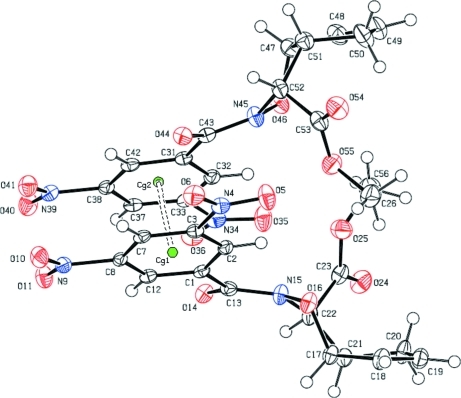
A view of a pair of independent molecules of (I) connected by π···π interactions (dashed lines) with the atom-labelling scheme. Displacement ellipsoids are drawn at the 50% probability level. H atoms are shown as spheres of arbitrary radius.

**Fig. 2.**
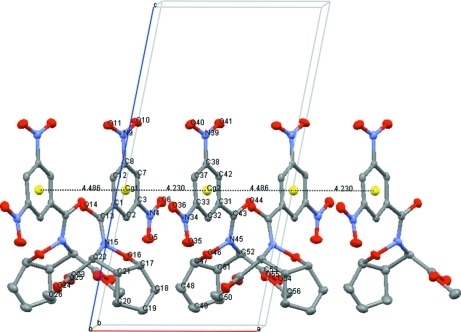
Part of the crystal structure of (I) viewed along the b axis. Dashed lines show Cg1-Cg2 (π···π) interactions along [100] direction and the respective distances are given in Å. H atoms are omitted for clarity.

**Fig. 3.**
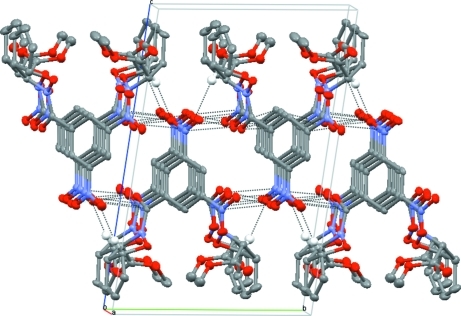
Part of the crystal structure of (I) viewed along the a axis. Dashed lines show CO···NO2, NO2···NO2 and C—H···O interactions along [010] direction. H atoms not involved in hydrogen bonding have been omitted for clarity.

## supplementary crystallographic information

### Comment

In organic synthesis, the usual techniques as NMR, mass or infra-red spectrometry and elemental analysis are often not enough for the unequivocally determination of a structure of a compound. When it is possible to crystallize desired compound, the X-ray crystallography is the ultimate analysis. 3,5-dinitrobenzoylation of 2-oxa-3-azabicyclo[3.3.0]oct-7-ene-4- carboxylate leaded to title compound (I) that was unambigously analysed by X-ray analysis.

The two independent molecules of the title compound (I) are coupled by π···π interactions of the 3,5-dinitrobenzoyl rings (Fig. 1) [C*g*1-C*g*2^iv^ = 4.2295 Å, symmetry code: (iv) 1 + *x*, *y*, *z*]. It is also possible to verify the existence of the three stereogenic centres of the same chirality in both molecules of the asymmetric unit. As the space group is centrosymmetric, a racemate is present in a crystal. No other stereoisomers of methyl 2-oxa-3-azabicyclo[3.3.0]oct-7-ene-4-carboxylate are obtained from the reported synthetic methodology (Sousa *et al.* 2008).

In the crystal structure, each pair of the molecules are linked by π··· π contacts between the 3,5-dinitrobenzoyl rings along [100] direction (Fig. 2) (C*g*1-C*g*2^iii^ = 4.4862 Å, C*g*2-C*g*1^iii^ = 4.4862 Å; symmetry code: (iii) *x*, *y*, *z*). Intermolecular interactions between carbonyl and nitro groups (distance C···O ≈ 3.0 Å), between nitro groups (distance N···O ≈ 3.0 Å) and C—H···O intermolecular hydrogen bonds (Table 1) generate an assembly by packing these chains along [010] direction (Fig. 3). Table 2 lists the interactions between aromatic rings (resulting in a π···π stacking assembly).

The carbonyl and nitro groups are very electronegative; as a result, the electronic density of the 3,5-dinitrobenzoyl rings is delocalized from the centre of π-system towards the electronegative O atoms. This delocalization origins from electrostatic intermolecular interactions between the oxygen atoms and the centre of the π-system (Table 3).

This analysis suggest that the most important intermolecular interactions in compound (I) are due to the 3,5-dinitrobenzoyl ring (including the nitro and carbonyl groups), which seems to be the main reason why compound (I) is a solid.

### Experimental

The title compound was synthesized from 2-oxa-3-azabicyclo[3.3.0]oct-7-ene-4-carboxylate as reported in literature (Sousa *et al.* 2008). Crystals were obtained from a slow evaporation of a dichlorometane/methanol/hexane solution of (I).

### Refinement

All H atoms were found in a difference Fourier map and placed in geometrically idealized and constrained to ride on their parent atoms [C—H = 0.93–0.98 Å and *U*_iso_(H) = 1.2 (1.5 for methyl groups) × *U*_eq_(C)].

### Crystal data

**Table:**

C_15_H_13_N_3_O_8_	*Z* = 4
*M**_r_* = 363.28	*F*(000) = 752
Triclinic, *P*1	*D*_x_ = 1.581 Mg m^−^^3^
Hall symbol: -P 1	Mo *K*α radiation, λ = 0.71069 Å
*a* = 8.7157 (3) Å	Cell parameters from 6065 reflections
*b* = 10.8269 (3) Å	θ = 2.4–26.0°
*c* = 17.0677 (5) Å	µ = 0.13 mm^−^^1^
α = 79.881 (1)°	*T* = 100 K
β = 77.773 (1)°	Prism, colourless
γ = 78.281 (1)°	0.26 × 0.23 × 0.1 mm
*V* = 1526.35 (8) Å^3^	

### Data collection

**Table:**

Bruker ApexII CCD area-detector diffractometer	6012 independent reflections
Radiation source: sealed tube	4564 reflections with *I* > 2σ(*I*)
graphite	*R*_int_ = 0.039
phi and ω scans	θ_max_ = 26.0°, θ_min_ = 1.9°
Absorption correction: multi-scan (*SADABS*; Bruker, 2007)	*h* = −10→10
*T*_min_ = 0.913, *T*_max_ = 0.99	*k* = −13→13
28142 measured reflections	*l* = 0→21

### Refinement

**Table:**

Refinement on *F*^2^	Primary atom site location: structure-invariant direct methods
Least-squares matrix: full	Secondary atom site location: difference Fourier map
*R*[*F*^2^ > 2σ(*F*^2^)] = 0.047	Hydrogen site location: inferred from neighbouring sites
*wR*(*F*^2^) = 0.124	H-atom parameters constrained
*S* = 1.05	*w* = 1/[σ^2^(*F*_o_^2^) + (0.0478*P*)^2^ + 1.4085*P*] where *P* = (*F*_o_^2^ + 2*F*_c_^2^)/3
6012 reflections	(Δ/σ)_max_ < 0.001
471 parameters	Δρ_max_ = 0.27 e Å^−^^3^
0 restraints	Δρ_min_ = −0.24 e Å^−^^3^

### Special details

**Table:**

Geometry. All e.s.d.'s (except the e.s.d. in the dihedral angle between two l.s. planes) are estimated using the full covariance matrix. The cell e.s.d.'s are taken into account individually in the estimation of e.s.d.'s in distances, angles and torsion angles; correlations between e.s.d.'s in cell parameters are only used when they are defined by crystal symmetry. An approximate (isotropic) treatment of cell e.s.d.'s is used for estimating e.s.d.'s involving l.s. planes.
Refinement. Refinement of *F*^2^ against ALL reflections. The weighted *R*-factor *wR* and goodness of fit *S* are based on *F*^2^, conventional *R*-factors *R* are based on *F*, with *F* set to zero for negative *F*^2^. The threshold expression of *F*^2^ > σ(*F*^2^) is used only for calculating *R*-factors(gt) *etc*. and is not relevant to the choice of reflections for refinement. *R*-factors based on *F*^2^ are statistically about twice as large as those based on *F*, and *R*– factors based on ALL data will be even larger.

### Fractional atomic coordinates and isotropic or equivalent isotropic displacement parameters (Å^2^)

**Table:**

	*x*	*y*	*z*	*U*_iso_*/*U*_eq_	
C1	0.9942 (2)	0.18394 (19)	0.38593 (13)	0.0155 (4)	
C2	1.0752 (2)	0.2818 (2)	0.34588 (13)	0.0166 (4)	
H2	1.0992	0.2956	0.2896	0.02*	
C3	1.1195 (2)	0.35814 (19)	0.39127 (13)	0.0160 (4)	
N4	1.2051 (2)	0.46174 (17)	0.34844 (11)	0.0195 (4)	
O5	1.2230 (2)	0.48129 (16)	0.27460 (10)	0.0313 (4)	
O6	1.25526 (19)	0.52204 (15)	0.38914 (10)	0.0246 (4)	
C7	1.0872 (2)	0.34329 (19)	0.47509 (13)	0.0159 (4)	
H7	1.1201	0.3946	0.5044	0.019*	
C8	1.0029 (2)	0.24721 (19)	0.51215 (13)	0.0152 (4)	
N9	0.9606 (2)	0.22901 (17)	0.60139 (11)	0.0187 (4)	
O10	1.0221 (2)	0.28525 (15)	0.63892 (10)	0.0267 (4)	
O11	0.8644 (2)	0.15906 (15)	0.63199 (10)	0.0269 (4)	
C12	0.9546 (2)	0.1685 (2)	0.47014 (13)	0.0165 (4)	
H12	0.8964	0.1059	0.4977	0.02*	
C13	0.9295 (3)	0.0975 (2)	0.34540 (13)	0.0176 (5)	
O14	0.81962 (18)	0.04407 (14)	0.38263 (9)	0.0207 (3)	
N15	0.9857 (2)	0.08830 (17)	0.26587 (11)	0.0189 (4)	
O16	1.13586 (18)	0.11886 (14)	0.22609 (9)	0.0213 (4)	
C17	1.2273 (3)	−0.0039 (2)	0.20221 (14)	0.0206 (5)	
H17	1.278	−0.0563	0.2458	0.025*	
C18	1.3444 (3)	0.0181 (2)	0.12586 (14)	0.0243 (5)	
H18	1.4385	0.0484	0.1223	0.029*	
C19	1.2969 (3)	−0.0111 (2)	0.06383 (14)	0.0259 (5)	
H19	1.3561	−0.0057	0.0116	0.031*	
C20	1.1382 (3)	−0.0538 (2)	0.08611 (14)	0.0264 (5)	
H20A	1.1434	−0.1343	0.0673	0.032*	
H20B	1.0571	0.0093	0.0635	0.032*	
C21	1.1035 (3)	−0.0680 (2)	0.17955 (13)	0.0200 (5)	
H21	1.1197	−0.1586	0.2016	0.024*	
C22	0.9406 (3)	−0.0004 (2)	0.22286 (13)	0.0201 (5)	
H22	0.8892	−0.0635	0.2626	0.024*	
C23	0.8278 (3)	0.0653 (2)	0.16619 (14)	0.0240 (5)	
O24	0.7600 (2)	0.00814 (17)	0.13342 (10)	0.0308 (4)	
O25	0.8140 (2)	0.19225 (16)	0.15572 (10)	0.0307 (4)	
C26	0.7075 (4)	0.2615 (3)	0.10190 (17)	0.0400 (7)	
H26A	0.7476	0.2395	0.0484	0.06*	
H26B	0.7009	0.3514	0.1007	0.06*	
H26C	0.6034	0.2394	0.121	0.06*	
C31	0.6061 (2)	0.36166 (19)	0.38716 (13)	0.0163 (4)	
C32	0.5616 (2)	0.2795 (2)	0.34533 (13)	0.0176 (5)	
H32	0.5863	0.2881	0.2891	0.021*	
C33	0.4798 (2)	0.1845 (2)	0.38893 (13)	0.0173 (5)	
N34	0.4341 (2)	0.09760 (17)	0.34395 (12)	0.0205 (4)	
O35	0.4812 (2)	0.10687 (17)	0.27083 (10)	0.0318 (4)	
O36	0.35044 (19)	0.02069 (15)	0.38273 (10)	0.0253 (4)	
C37	0.4382 (2)	0.16728 (19)	0.47197 (13)	0.0175 (5)	
H37	0.3799	0.1049	0.4997	0.021*	
C38	0.4885 (2)	0.2484 (2)	0.51171 (13)	0.0164 (4)	
N39	0.4501 (2)	0.23228 (17)	0.60065 (11)	0.0196 (4)	
O40	0.3526 (2)	0.16400 (16)	0.63497 (10)	0.0290 (4)	
O41	0.5182 (2)	0.28796 (15)	0.63533 (10)	0.0268 (4)	
C42	0.5721 (2)	0.3436 (2)	0.47166 (13)	0.0175 (5)	
H42	0.6055	0.3951	0.5007	0.021*	
C43	0.7027 (3)	0.4644 (2)	0.34872 (13)	0.0179 (5)	
O44	0.78205 (18)	0.50231 (14)	0.38786 (9)	0.0209 (3)	
N45	0.7092 (2)	0.50775 (18)	0.26884 (11)	0.0209 (4)	
O46	0.59327 (19)	0.49161 (14)	0.22645 (9)	0.0234 (4)	
C47	0.5203 (3)	0.6231 (2)	0.19852 (14)	0.0242 (5)	
H47	0.4338	0.6587	0.2395	0.029*	
C48	0.4685 (3)	0.6268 (2)	0.12065 (15)	0.0294 (6)	
H48	0.3777	0.5984	0.1157	0.035*	
C49	0.5688 (3)	0.6761 (3)	0.05900 (16)	0.0346 (6)	
H49	0.556	0.686	0.0054	0.042*	
C50	0.7046 (3)	0.7143 (3)	0.08449 (15)	0.0358 (6)	
H50A	0.7137	0.8021	0.0628	0.043*	
H50B	0.8045	0.6598	0.0666	0.043*	
C51	0.6601 (3)	0.6973 (2)	0.17758 (14)	0.0241 (5)	
H51	0.6251	0.7809	0.1962	0.029*	
C52	0.7859 (3)	0.6152 (2)	0.22667 (14)	0.0221 (5)	
H52	0.8012	0.664	0.2668	0.026*	
C53	0.9452 (3)	0.5754 (2)	0.17384 (14)	0.0241 (5)	
O54	1.0372 (2)	0.64757 (16)	0.14466 (11)	0.0317 (4)	
O55	0.9688 (2)	0.45359 (16)	0.16190 (10)	0.0292 (4)	
C56	1.1169 (3)	0.4105 (3)	0.10927 (16)	0.0362 (6)	
H56A	1.1234	0.4644	0.058	0.054*	
H56B	1.1202	0.3244	0.1012	0.054*	
H56C	1.2052	0.4142	0.1337	0.054*	

### Atomic displacement parameters (Å^2^)

**Table:**

	*U*^11^	*U*^22^	*U*^33^	*U*^12^	*U*^13^	*U*^23^
C1	0.0128 (10)	0.0146 (10)	0.0202 (11)	0.0019 (8)	−0.0055 (8)	−0.0069 (8)
C2	0.0151 (11)	0.0174 (11)	0.0166 (11)	0.0011 (8)	−0.0038 (8)	−0.0042 (8)
C3	0.0127 (10)	0.0142 (10)	0.0211 (12)	−0.0016 (8)	−0.0034 (9)	−0.0026 (8)
N4	0.0183 (10)	0.0174 (9)	0.0239 (11)	−0.0043 (8)	−0.0045 (8)	−0.0035 (8)
O5	0.0433 (11)	0.0351 (10)	0.0194 (9)	−0.0212 (8)	−0.0033 (8)	−0.0003 (7)
O6	0.0266 (9)	0.0219 (8)	0.0310 (9)	−0.0100 (7)	−0.0095 (7)	−0.0073 (7)
C7	0.0122 (10)	0.0154 (10)	0.0217 (12)	0.0023 (8)	−0.0071 (9)	−0.0072 (9)
C8	0.0121 (10)	0.0178 (11)	0.0154 (11)	0.0025 (8)	−0.0043 (8)	−0.0050 (8)
N9	0.0192 (10)	0.0189 (9)	0.0183 (10)	−0.0016 (8)	−0.0046 (8)	−0.0035 (8)
O10	0.0353 (10)	0.0287 (9)	0.0213 (9)	−0.0083 (8)	−0.0118 (7)	−0.0063 (7)
O11	0.0319 (9)	0.0299 (9)	0.0204 (9)	−0.0129 (8)	−0.0024 (7)	−0.0017 (7)
C12	0.0124 (10)	0.0156 (10)	0.0215 (12)	−0.0003 (8)	−0.0035 (9)	−0.0042 (9)
C13	0.0181 (11)	0.0160 (11)	0.0199 (12)	−0.0003 (9)	−0.0079 (9)	−0.0037 (9)
O14	0.0179 (8)	0.0228 (8)	0.0234 (8)	−0.0070 (7)	−0.0018 (6)	−0.0072 (7)
N15	0.0194 (10)	0.0240 (10)	0.0168 (10)	−0.0108 (8)	−0.0014 (8)	−0.0067 (8)
O16	0.0215 (8)	0.0247 (8)	0.0202 (8)	−0.0118 (7)	0.0023 (6)	−0.0077 (7)
C17	0.0226 (12)	0.0226 (11)	0.0194 (12)	−0.0071 (9)	−0.0030 (9)	−0.0077 (9)
C18	0.0251 (13)	0.0274 (12)	0.0219 (12)	−0.0099 (10)	0.0008 (10)	−0.0072 (10)
C19	0.0290 (13)	0.0275 (13)	0.0198 (12)	−0.0055 (10)	0.0008 (10)	−0.0055 (10)
C20	0.0270 (13)	0.0387 (14)	0.0171 (12)	−0.0081 (11)	−0.0025 (10)	−0.0124 (10)
C21	0.0233 (12)	0.0208 (11)	0.0191 (12)	−0.0069 (9)	−0.0054 (9)	−0.0060 (9)
C22	0.0250 (12)	0.0219 (11)	0.0178 (12)	−0.0112 (9)	−0.0029 (9)	−0.0075 (9)
C23	0.0229 (12)	0.0285 (13)	0.0221 (12)	−0.0048 (10)	−0.0019 (10)	−0.0103 (10)
O24	0.0285 (9)	0.0382 (10)	0.0328 (10)	−0.0059 (8)	−0.0123 (8)	−0.0159 (8)
O25	0.0382 (10)	0.0264 (9)	0.0315 (10)	−0.0015 (8)	−0.0165 (8)	−0.0067 (7)
C26	0.0500 (18)	0.0405 (16)	0.0317 (15)	0.0081 (13)	−0.0213 (13)	−0.0123 (12)
C31	0.0126 (10)	0.0148 (10)	0.0217 (12)	−0.0002 (8)	−0.0044 (9)	−0.0038 (9)
C32	0.0153 (11)	0.0186 (11)	0.0186 (12)	0.0011 (8)	−0.0052 (9)	−0.0041 (9)
C33	0.0144 (10)	0.0153 (10)	0.0240 (12)	−0.0002 (8)	−0.0060 (9)	−0.0073 (9)
N34	0.0193 (10)	0.0182 (10)	0.0269 (11)	−0.0023 (8)	−0.0086 (8)	−0.0072 (8)
O35	0.0441 (11)	0.0366 (10)	0.0211 (10)	−0.0182 (8)	−0.0053 (8)	−0.0085 (7)
O36	0.0254 (9)	0.0199 (8)	0.0338 (10)	−0.0093 (7)	−0.0069 (7)	−0.0043 (7)
C37	0.0135 (10)	0.0129 (10)	0.0257 (12)	−0.0004 (8)	−0.0044 (9)	−0.0030 (9)
C38	0.0124 (10)	0.0177 (11)	0.0181 (11)	0.0016 (8)	−0.0035 (8)	−0.0037 (9)
N39	0.0182 (10)	0.0186 (9)	0.0213 (10)	−0.0014 (8)	−0.0022 (8)	−0.0051 (8)
O40	0.0308 (9)	0.0296 (9)	0.0262 (9)	−0.0129 (8)	0.0036 (7)	−0.0048 (7)
O41	0.0300 (9)	0.0285 (9)	0.0248 (9)	−0.0079 (7)	−0.0062 (7)	−0.0069 (7)
C42	0.0150 (11)	0.0146 (10)	0.0246 (12)	0.0013 (8)	−0.0082 (9)	−0.0062 (9)
C43	0.0151 (11)	0.0158 (11)	0.0236 (12)	−0.0016 (8)	−0.0039 (9)	−0.0057 (9)
O44	0.0214 (8)	0.0200 (8)	0.0247 (9)	−0.0068 (7)	−0.0078 (7)	−0.0036 (6)
N45	0.0250 (10)	0.0241 (10)	0.0184 (10)	−0.0124 (8)	−0.0073 (8)	−0.0019 (8)
O46	0.0310 (9)	0.0233 (8)	0.0221 (9)	−0.0116 (7)	−0.0134 (7)	−0.0011 (7)
C47	0.0253 (12)	0.0229 (12)	0.0253 (13)	−0.0062 (10)	−0.0062 (10)	−0.0012 (10)
C48	0.0358 (14)	0.0269 (13)	0.0284 (14)	−0.0073 (11)	−0.0140 (11)	0.0001 (10)
C49	0.0433 (16)	0.0377 (15)	0.0226 (14)	−0.0041 (12)	−0.0123 (12)	0.0005 (11)
C50	0.0349 (15)	0.0435 (16)	0.0252 (14)	−0.0083 (12)	−0.0076 (11)	0.0098 (12)
C51	0.0262 (13)	0.0217 (12)	0.0249 (13)	−0.0068 (10)	−0.0045 (10)	−0.0016 (9)
C52	0.0304 (13)	0.0204 (11)	0.0183 (12)	−0.0102 (10)	−0.0062 (10)	−0.0017 (9)
C53	0.0280 (13)	0.0254 (12)	0.0212 (12)	−0.0086 (10)	−0.0089 (10)	0.0007 (10)
O54	0.0287 (10)	0.0324 (10)	0.0342 (10)	−0.0134 (8)	−0.0028 (8)	0.0008 (8)
O55	0.0315 (10)	0.0266 (9)	0.0284 (9)	−0.0067 (7)	−0.0002 (8)	−0.0059 (7)
C56	0.0370 (15)	0.0363 (15)	0.0307 (15)	−0.0046 (12)	0.0028 (12)	−0.0051 (12)

### Geometric parameters (Å, °)

**Table:**

C1—C2	1.390 (3)	C31—C32	1.390 (3)
C1—C12	1.393 (3)	C31—C42	1.396 (3)
C1—C13	1.510 (3)	C31—C43	1.505 (3)
C2—C3	1.384 (3)	C32—C33	1.388 (3)
C2—H2	0.93	C32—H32	0.93
C3—C7	1.385 (3)	C33—C37	1.375 (3)
C3—N4	1.473 (3)	C33—N34	1.473 (3)
N4—O5	1.222 (2)	N34—O35	1.221 (2)
N4—O6	1.226 (2)	N34—O36	1.230 (2)
C7—C8	1.380 (3)	C37—C38	1.384 (3)
C7—H7	0.93	C37—H37	0.93
C8—C12	1.380 (3)	C38—C42	1.381 (3)
C8—N9	1.477 (3)	C38—N39	1.470 (3)
N9—O11	1.217 (2)	N39—O41	1.218 (2)
N9—O10	1.218 (2)	N39—O40	1.226 (2)
C12—H12	0.93	C42—H42	0.93
C13—O14	1.222 (3)	C43—O44	1.225 (3)
C13—N15	1.356 (3)	C43—N45	1.355 (3)
N15—O16	1.416 (2)	N45—O46	1.414 (2)
N15—C22	1.463 (3)	N45—C52	1.461 (3)
O16—C17	1.480 (3)	O46—C47	1.476 (3)
C17—C18	1.491 (3)	C47—C48	1.483 (3)
C17—C21	1.539 (3)	C47—C51	1.540 (3)
C17—H17	0.98	C47—H47	0.98
C18—C19	1.323 (3)	C48—C49	1.326 (4)
C18—H18	0.93	C48—H48	0.93
C19—C20	1.499 (3)	C49—C50	1.498 (4)
C19—H19	0.93	C49—H49	0.93
C20—C21	1.545 (3)	C50—C51	1.541 (3)
C20—H20A	0.97	C50—H50A	0.97
C20—H20B	0.97	C50—H50B	0.97
C21—C22	1.556 (3)	C51—C52	1.557 (3)
C21—H21	0.98	C51—H51	0.98
C22—C23	1.512 (3)	C52—C53	1.513 (3)
C22—H22	0.98	C52—H52	0.98
C23—O24	1.206 (3)	C53—O54	1.204 (3)
C23—O25	1.338 (3)	C53—O55	1.338 (3)
O25—C26	1.451 (3)	O55—C56	1.450 (3)
C26—H26A	0.96	C56—H56A	0.96
C26—H26B	0.96	C56—H56B	0.96
C26—H26C	0.96	C56—H56C	0.96
			
C2—C1—C12	119.33 (19)	C32—C31—C42	119.20 (19)
C2—C1—C13	125.21 (19)	C32—C31—C43	124.9 (2)
C12—C1—C13	115.20 (19)	C42—C31—C43	115.68 (19)
C3—C2—C1	118.7 (2)	C33—C32—C31	118.8 (2)
C3—C2—H2	120.6	C33—C32—H32	120.6
C1—C2—H2	120.6	C31—C32—H32	120.6
C2—C3—C7	123.71 (19)	C37—C33—C32	123.6 (2)
C2—C3—N4	118.47 (19)	C37—C33—N34	118.10 (19)
C7—C3—N4	117.82 (18)	C32—C33—N34	118.30 (19)
O5—N4—O6	124.19 (18)	O35—N34—O36	124.40 (18)
O5—N4—C3	117.94 (17)	O35—N34—C33	118.04 (18)
O6—N4—C3	117.87 (18)	O36—N34—C33	117.56 (18)
C8—C7—C3	115.49 (19)	C33—C37—C38	115.97 (19)
C8—C7—H7	122.3	C33—C37—H37	122
C3—C7—H7	122.3	C38—C37—H37	122
C12—C8—C7	123.4 (2)	C42—C38—C37	123.0 (2)
C12—C8—N9	118.47 (18)	C42—C38—N39	118.83 (19)
C7—C8—N9	118.09 (18)	C37—C38—N39	118.16 (19)
O11—N9—O10	124.79 (19)	O41—N39—O40	124.37 (19)
O11—N9—C8	116.85 (17)	O41—N39—C38	117.38 (18)
O10—N9—C8	118.35 (18)	O40—N39—C38	118.24 (18)
C8—C12—C1	119.23 (19)	C38—C42—C31	119.3 (2)
C8—C12—H12	120.4	C38—C42—H42	120.3
C1—C12—H12	120.4	C31—C42—H42	120.3
O14—C13—N15	120.63 (19)	O44—C43—N45	120.51 (19)
O14—C13—C1	119.92 (19)	O44—C43—C31	120.4 (2)
N15—C13—C1	119.24 (18)	N45—C43—C31	118.93 (19)
C13—N15—O16	122.19 (17)	C43—N45—O46	121.93 (17)
C13—N15—C22	123.37 (17)	C43—N45—C52	123.86 (18)
O16—N15—C22	109.47 (15)	O46—N45—C52	109.63 (16)
N15—O16—C17	103.45 (14)	N45—O46—C47	103.91 (15)
O16—C17—C18	110.40 (18)	O46—C47—C48	109.52 (19)
O16—C17—C21	104.41 (17)	O46—C47—C51	104.18 (18)
C18—C17—C21	105.22 (18)	C48—C47—C51	105.38 (19)
O16—C17—H17	112.1	O46—C47—H47	112.4
C18—C17—H17	112.1	C48—C47—H47	112.4
C21—C17—H17	112.1	C51—C47—H47	112.4
C19—C18—C17	111.1 (2)	C49—C48—C47	111.0 (2)
C19—C18—H18	124.5	C49—C48—H48	124.5
C17—C18—H18	124.5	C47—C48—H48	124.5
C18—C19—C20	113.4 (2)	C48—C49—C50	113.1 (2)
C18—C19—H19	123.3	C48—C49—H49	123.4
C20—C19—H19	123.3	C50—C49—H49	123.4
C19—C20—C21	103.39 (18)	C49—C50—C51	103.6 (2)
C19—C20—H20A	111.1	C49—C50—H50A	111
C21—C20—H20A	111.1	C51—C50—H50A	111
C19—C20—H20B	111.1	C49—C50—H50B	111
C21—C20—H20B	111.1	C51—C50—H50B	111
H20A—C20—H20B	109	H50A—C50—H50B	109
C17—C21—C20	105.39 (18)	C47—C51—C50	105.2 (2)
C17—C21—C22	104.16 (17)	C47—C51—C52	104.17 (18)
C20—C21—C22	118.67 (19)	C50—C51—C52	117.9 (2)
C17—C21—H21	109.4	C47—C51—H51	109.7
C20—C21—H21	109.4	C50—C51—H51	109.7
C22—C21—H21	109.4	C52—C51—H51	109.7
N15—C22—C23	112.66 (18)	N45—C52—C53	113.37 (19)
N15—C22—C21	103.44 (17)	N45—C52—C51	103.52 (18)
C23—C22—C21	113.97 (18)	C53—C52—C51	112.90 (19)
N15—C22—H22	108.9	N45—C52—H52	109
C23—C22—H22	108.9	C53—C52—H52	108.9
C21—C22—H22	108.9	C51—C52—H52	109
O24—C23—O25	124.3 (2)	O54—C53—O55	124.6 (2)
O24—C23—C22	122.9 (2)	O54—C53—C52	123.0 (2)
O25—C23—C22	112.79 (19)	O55—C53—C52	112.42 (19)
C23—O25—C26	115.69 (19)	C53—O55—C56	115.30 (19)
O25—C26—H26A	109.5	O55—C56—H56A	109.5
O25—C26—H26B	109.5	O55—C56—H56B	109.5
H26A—C26—H26B	109.5	H56A—C56—H56B	109.5
O25—C26—H26C	109.5	O55—C56—H56C	109.5
H26A—C26—H26C	109.5	H56A—C56—H56C	109.5
H26B—C26—H26C	109.5	H56B—C56—H56C	109.5
			
C12—C1—C2—C3	2.4 (3)	C42—C31—C32—C33	2.2 (3)
C13—C1—C2—C3	176.23 (19)	C43—C31—C32—C33	176.5 (2)
C1—C2—C3—C7	−0.3 (3)	C31—C32—C33—C37	0.6 (3)
C1—C2—C3—N4	−179.91 (18)	C31—C32—C33—N34	−179.63 (18)
C2—C3—N4—O5	5.2 (3)	C37—C33—N34—O35	−174.67 (19)
C7—C3—N4—O5	−174.49 (19)	C32—C33—N34—O35	5.6 (3)
C2—C3—N4—O6	−174.10 (19)	C37—C33—N34—O36	5.6 (3)
C7—C3—N4—O6	6.2 (3)	C32—C33—N34—O36	−174.15 (19)
C2—C3—C7—C8	−1.4 (3)	C32—C33—C37—C38	−2.4 (3)
N4—C3—C7—C8	178.24 (18)	N34—C33—C37—C38	177.81 (18)
C3—C7—C8—C12	1.0 (3)	C33—C37—C38—C42	1.5 (3)
C3—C7—C8—N9	−178.24 (18)	C33—C37—C38—N39	−178.77 (18)
C12—C8—N9—O11	−11.2 (3)	C42—C38—N39—O41	−13.0 (3)
C7—C8—N9—O11	168.04 (19)	C37—C38—N39—O41	167.26 (19)
C12—C8—N9—O10	169.49 (19)	C42—C38—N39—O40	167.13 (19)
C7—C8—N9—O10	−11.2 (3)	C37—C38—N39—O40	−12.6 (3)
C7—C8—C12—C1	1.1 (3)	C37—C38—C42—C31	1.2 (3)
N9—C8—C12—C1	−179.67 (18)	N39—C38—C42—C31	−178.54 (18)
C2—C1—C12—C8	−2.8 (3)	C32—C31—C42—C38	−3.0 (3)
C13—C1—C12—C8	−177.25 (18)	C43—C31—C42—C38	−177.91 (19)
C2—C1—C13—O14	−156.4 (2)	C32—C31—C43—O44	−154.8 (2)
C12—C1—C13—O14	17.7 (3)	C42—C31—C43—O44	19.7 (3)
C2—C1—C13—N15	18.3 (3)	C32—C31—C43—N45	20.6 (3)
C12—C1—C13—N15	−167.64 (19)	C42—C31—C43—N45	−164.86 (19)
O14—C13—N15—O16	−163.04 (19)	O44—C43—N45—O46	−164.20 (19)
C1—C13—N15—O16	22.3 (3)	C31—C43—N45—O46	20.4 (3)
O14—C13—N15—C22	−10.6 (3)	O44—C43—N45—C52	−10.7 (3)
C1—C13—N15—C22	174.73 (19)	C31—C43—N45—C52	173.9 (2)
C13—N15—O16—C17	116.3 (2)	C43—N45—O46—C47	118.3 (2)
C22—N15—O16—C17	−39.5 (2)	C52—N45—O46—C47	−38.5 (2)
N15—O16—C17—C18	148.95 (18)	N45—O46—C47—C48	148.66 (18)
N15—O16—C17—C21	36.3 (2)	N45—O46—C47—C51	36.3 (2)
O16—C17—C18—C19	−105.7 (2)	O46—C47—C48—C49	−103.5 (2)
C21—C17—C18—C19	6.4 (3)	C51—C47—C48—C49	8.1 (3)
C17—C18—C19—C20	1.9 (3)	C47—C48—C49—C50	−0.2 (3)
C18—C19—C20—C21	−9.1 (3)	C48—C49—C50—C51	−7.8 (3)
O16—C17—C21—C20	104.78 (19)	O46—C47—C51—C50	103.0 (2)
C18—C17—C21—C20	−11.5 (2)	C48—C47—C51—C50	−12.3 (3)
O16—C17—C21—C22	−20.9 (2)	O46—C47—C51—C52	−21.7 (2)
C18—C17—C21—C22	−137.14 (19)	C48—C47—C51—C52	−137.0 (2)
C19—C20—C21—C17	12.2 (2)	C49—C50—C51—C47	12.0 (3)
C19—C20—C21—C22	128.3 (2)	C49—C50—C51—C52	127.5 (2)
C13—N15—C22—C23	106.4 (2)	C43—N45—C52—C53	104.7 (2)
O16—N15—C22—C23	−98.2 (2)	O46—N45—C52—C53	−99.0 (2)
C13—N15—C22—C21	−130.1 (2)	C43—N45—C52—C51	−132.6 (2)
O16—N15—C22—C21	25.4 (2)	O46—N45—C52—C51	23.7 (2)
C17—C21—C22—N15	−1.6 (2)	C47—C51—C52—N45	−0.1 (2)
C20—C21—C22—N15	−118.4 (2)	C50—C51—C52—N45	−116.2 (2)
C17—C21—C22—C23	121.1 (2)	C47—C51—C52—C53	122.8 (2)
C20—C21—C22—C23	4.3 (3)	C50—C51—C52—C53	6.7 (3)
N15—C22—C23—O24	−169.9 (2)	N45—C52—C53—O54	−168.0 (2)
C21—C22—C23—O24	72.7 (3)	C51—C52—C53—O54	74.7 (3)
N15—C22—C23—O25	11.2 (3)	N45—C52—C53—O55	13.8 (3)
C21—C22—C23—O25	−106.3 (2)	C51—C52—C53—O55	−103.5 (2)
O24—C23—O25—C26	1.0 (3)	O54—C53—O55—C56	−0.4 (3)
C22—C23—O25—C26	179.9 (2)	C52—C53—O55—C56	177.8 (2)

### Hydrogen-bond geometry (Å, °)

**Table:**

*D*—H···*A*	*D*—H	H···*A*	*D*···*A*	*D*—H···*A*
C17—H17···O11^i^	0.98	2.40	3.064 (3)	124
C47—H47···O41^ii^	0.98	2.45	3.083 (3)	122

Symmetry codes: (i) −*x*+2, −*y*, −*z*+1; (ii) −*x*+1, −*y*+1, −*z*+1.

### Table 2 Table 2. Geometric parameters of π···π contacts (Å, °)

**Table:**

Cg X···Cg Y	Cg···Cg	α	β
Cg1···Cg2	4.4862	0.49 (9)	39.43
Cg1···Cg2iii	4.2295	0.49 (9)	39.50
Cg2···Cg1iv	4.2296	0.49 (9)	39.15
Cg2···Cg1	4.4862	0.49 (9)	39.08

Symmetry codes: (iii) 1+x, y, z; (iv) -1+x, y, z. Cg1 and Cg2 are the centroids of the rings defined by C1, C2, C3, C7, C8, C12 and C31, C32, C33, C37, C38 C42, respectively.

### Table 3 Table 3. Geometric parameters of Y—X···Cg (π···ring) contacts (Å, °)

**Table:**

Y—X···Cg	X···Cg	Y—X···Cg	Y···Cg
N4—O6···Cg2iii	3.3345 (18)	88.47 (12)	3.522 (2)
C13—O14···Cg2	3.2009 (18)	106.47 (13)	3.736 (3)
N34—O36···Cg1iv	3.4450 (18)	91.01 (12)	3.678 (2)
C43—O44···Cg1	3.1434 (18)	104.52 (13)	3.649 (3)

Symmetry codes: (iii) 1+x, y, z; (iv) -1+x, y, z. Cg1 and Cg2 are the centroids of the rings defined by C1, C2, C3, C7, C8, C12 and C31, C32, C33, C37, C38 C42, respectively.
